# Inequity in Health Among People Living With Alzheimer's Disease and Related Dementias in Adult Day Centers

**DOI:** 10.1093/geroni/igab046.1275

**Published:** 2021-12-17

**Authors:** Jonelle Boafo, Gary Yu, Bei Wu, Abraham Brody, Tina Sadarangani

**Affiliations:** 1 Rory Meyers College of Nursing, New York, New York, United States; 2 NYU Rory Meyers College of nursing, New York, New York, United States; 3 New York University, New York, New York, United States; 4 NYU Hartford Institute for Geriatric Nursing, New York, New York, United States; 5 New York University Rory Meyers College of Nursing, New York University Rory Meyers College of Nursing, New York, United States

## Abstract

In adult day centers (ADCs), 58% of clients identify as racial/ethnic minorities, and at least 30% have Alzheimer’s Disease and related dementias (ADRD). ADCs offer culturally and linguistically congruent care to clients, making them well-positioned to address potential health disparities affecting persons with ADRD. We used data from 53 California ADCs (n=3,053) to identify differences in clinical characteristics among ADC clients’ with ADRD based on demographics such as race and English proficiency. We found that, when compared to their respective counterparts, a significantly greater proportion of racial/ethnic minorities and non-English speakers (p<.001) had 5 or more chronic conditions in addition to ADRD. We noted considerable missing data on race, likely because ADCs in California are not mandated to report data on race/ethnicity. In order to identify inequities in care within this complex population, social determinants of health, including race, must be a standard component of client assessment.

